# Remission Period in Children With Newly Diagnosed Type 1 Diabetes During the COVID-19 Pandemic-Results From the DPV Registry

**DOI:** 10.1155/pedi/9903467

**Published:** 2025-05-15

**Authors:** Valentina Lahn, Sascha R. Tittel, Ute Ohlenschläger, Clemens Kamrath, Johanna Hammersen, Renata Gellai, Kirsten Mönkemöller, Axel Dost, Heike Bartelt, Reinhard W. Holl

**Affiliations:** ^1^Department of Pediatrics, Altona Children's Hospital, Hamburg, Germany; ^2^Institute of Epidemiology and Medical Biometry, ZIBMT, University of Ulm, Ulm, Germany; ^3^German Center for Diabetes Research (DZD), Munich-Neuherberg, Germany; ^4^Center of Children and Adolescent, Friedrich-Ebert-Hospital, Neumuenster, Germany; ^5^Center of Child and Adolescent Medicine, University of Freiburg, Freiburg, Germany; ^6^Department of Pediatrics, University Hospital Erlangen, Erlangen, Germany; ^7^Department of Pediatrics, Klinik Favoriten, Vienna, Austria; ^8^Department of Pediatrics, Children's Hospital, Amsterdamer Street, Koeln, Germany; ^9^Department of Pediatrics, University Hospital Jena, Jena, Germany; ^10^Center of Child and Adolescent Medicine, University Hospital Leipzig, Leipzig, Germany

## Abstract

To investigate whether the remission period in type 1 diabetes, as measured by insulin-dose adjusted A1c (IDAA1C), was affected by the COVID-19 pandemic. Data from 7603 children and adolescents with type 1 diabetes from the prospective diabetes follow-up (DPV) registry were available. We compared two time periods of diabetes onset, 2020/2021 vs. 2018/2019. IDAA1C and remission prevalence (IDAA1c < 9%) were analyzed using logistic and linear regression models adjusted for age groups (0.5–<6, 6–<12, and 12–<18 years), sex, diabetic ketoacidosis (DKA) at onset, use of continuous glucose monitoring (CGM) systems, insulin pumps, sensor-augmented pumps (SAPs) or automated insulin delivery (AID) systems, BMI categories (<90. percentile of BMI, 90. −<97. percentile of BMI, 97. −<99.5 percentile of BMI, > = 99.5 percentile of BMI) and immigrant background. Data from three time periods were analyzed: 3–5 months, 6–10 months, and 11–13 months after diagnosis of type 1 diabetes. Compared to the prepandemic period, during the COVID-19 pandemic adjusted IDAA1C was significantly higher at 3–5 months after diagnosis (mean estimated differences 0.26 [95% confidence interval 0.17; 0.35], *p*  < 0.001), but not at 6–10 months and 11–13 months after diagnosis (mean estimated difference 0.08 [−0.01; 0.17], *p*=0.07; and –0.03 [−0.12; 0.07], *p*=0.60), reflecting a lower percentage of patients in remission at 3–5 months. Reasons may be changes in autoimmune progression during the pandemic, lack of physical activities, increased stress or psychological burden, or altered access to care with delayed diagnosis of diabetes. Underlying causes need to be evaluated in future studies.

## 1. Introduction

An increase in the incidence of type 1 diabetes in children during the COVID-19 pandemic in Germany [1, 2], as well as in other parts of the world [3], has been reported [4]. However, the direct or indirect role of SARS-CoV-2, the pathogen that causes COVID-19, for this increase in diabetes incidence is not known. It has been shown recently that SARS-CoV-2 infection was associated with the development of islet autoantibodies in young children with a high genetic risk for type 1 diabetes [5]. In contrast, cohort studies found no evidence for a causative role of SARS-CoV-2 infection in the development of type 1 diabetes autoimmunity [6–9]. On the other hand, indirect effects of the pandemic may have also contributed to the increase in diabetes incidence [3, 9].

During the pandemic, in addition to an increasing incidence of type-1 diabetes, changes in the activity of the autoimmune process were reported as well. Indirect reasons that affected the remission of type-1 diabetes during the pandemic may be a lack of physical activities, increased stress from the pandemic itself or psychological burden, and altered access to medical care. Social contacts, child health care practices, and the behavior of families abruptly changed during the COVID-19 pandemic, due to the COVID-19 control efforts [10].

According to the hygiene hypothesis, there is an inverse association between the frequency of infectious diseases in early life and the occurrence of autoimmune diseases. The biodiversity hypothesis, an extension of the hygiene hypothesis, states that decreased biodiversity in the external and internal exposure increases the risk of immune-mediated diseases [10].

As another indirect effect during the pandemic, an elevated BMI during early childhood may exacerbate islet autoimmunity [11, 12].

Children with the onset of type 1 diabetes during the COVID-19 pandemic had a significantly higher daily insulin requirement after initiation of therapy. This could suggest a more rapid autoimmune destruction of beta cells during the pandemic [4].

The aim of this study is to investigate whether remission, as reflected by insulin-dose adjusted A1c (IDAA1C) [13, 14], was affected by the COVID-19 pandemic.

## 2. Methods

### 2.1. Study Design

This study was based on data from the diabetes follow-up (DPV) registry. The DPV registry incorporates pseudonymized, standardized, prospective data from routine diabetes care at centers in Germany, Austria, Switzerland, and Luxembourg. Data are transmitted twice a year for central validation and analysis. For optimal data validity, inconsistent data are reported back to participating centers, corrected if necessary, and re-entered into the database as previously described [15]. Corrected data are completely anonymized and integrated into the cumulative DPV registry. The DPV initiative, as well as the analyses of anonymized data, have been approved by the ethics committee at the University of Ulm (approval number 314/21). Participating centers obtained local data protection approval.

Data from 7603 children and adolescents with type 1 diabetes from the prospective DPV registry were available. We compared two time periods of diabetes onset, 2020/2021 vs. 2018/2019. IDAA1C value and prevalence of remission (IDAA1c < 9%) were analyzed using logistic and linear regression models adjusted for age groups (0.5–<6, 6–<12, and 12–<18 years), sex, diabetic ketoacidosis (DKA), use of continuous glucose monitoring (CGM) systems, insulin pump therapy, sensor-augmented pumps (SAPs), automated insulin delivery (AID) systems, BMI category and immigrant background. Patient data were analyzed at 3 time periods (3–5 months, 6–10 months, and 11–13 months) after diagnosis of type 1 diabetes.

### 2.2. Variables

Demographic and therapeutic characteristics of patients included biological sex, DKA, age at diabetes diagnosis, age and duration of diabetes at each contact, use of CGM systems, insulin pump therapy, SAPs, and AID systems, height, weight and body-mass index (BMI). The BMI is defined as the body weight divided by the square of the body height, and expressed in units of kg/m2. BMI-standard deviation scores (SDSs) were calculated according to the German Health Interview and Examination Survey for Children and Adolescents (KiGGS) study [16].

In accordance with the definition of the European Union/European Commission, an immigrant background was assigned if the patient or at least one parent was born outside of Germany, Austria, Switzerland, or Luxembourg.

Main outcomes were daily insulin dose (in units per kg body weight), the insulin-dose adjusted HbA1c (IDAA1C: HbA1c [%] + 4 × insulin dose [IU/kg/d]), and the remission status (IDAA1C ≤ 9%) [8]. The IDAA1C reflects residual beta cell function, including glycaemic control as well as daily insulin dose. A calculated IDAA1C ≤9 defines partial remission [14].

Rate of remission and IDAA1C were analyzed using logistic and linear regression models adjusted for age groups (0.5 < 6, 6–<12, and 12–<18 years), sex, DKA, insulin pump therapy, CGM systems, SAPs, AID systems, BMI category and immigrant background.

### 2.3. Statistical Methods

Demographic characteristics were compared between the two time periods of diabetes onset and presented as median with interquartile range (IQR) or percentage. Locally measured HbA1c values were mathematically standardized to the Diabetes Control and Complications Trial (DCCT) reference range (4.05%–6.05%) using the “multiple of the mean” transformation method (MOM-DCCT) [17].

For the main outcomes IDAA1C and remission, we calculated hierarchical linear and logistic regression models, respectively, adjusting for age, sex, DKA, BMI-SDS category (<90, 90−<97, 97−<99.5 and ≥99.5 percentile), immigrant background, CGM, insulin pump therapy, SAPs, and AID. A random intercept for treatment site was added (subject-specific model) to account for between-center heterogeneity. We used a diagonal covariance structure, which implies zero covariance between the random coefficients. Outcomes are presented as least-square means with 95% confidence intervals. A two-sided *p*-value <0.05 was considered significant. All analyses were implemented using SAS 9.4 (build TS1M7) on a Windows Server 2019 mainframe.

## 3. Results

**Figure 1:**
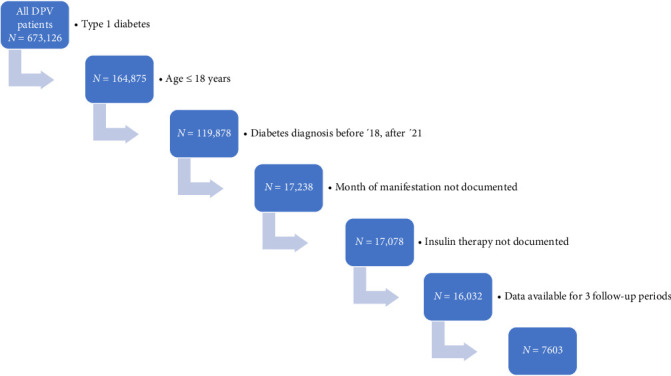
Flow chart to visualize patient selection by showing the number of excluded patients.

We included 7603 children and adolescents in the study (Figure 1). Median age at diagnosis of the whole cohort was 9.4 (IQR 5.6; 12.5) years, 55.0% (*N* = 4159) were male, 27.1% (*N* = 2060) had an immigrant background, and 29.3% (*N* = 2228) used an insulin pump within the first 10 days after manifestation, 44.6% used a CGM system (*N* = 3390), 12.2% used a SAP (*N* = 927) and 21.2% used an AID system (*N* = 1611). Further demographics are shown in Table 1.

About 45.5% of the patients (*N* = 3459) were diagnosed with type 1 diabetes before the occurrence of COVID-19 from 2018 to 2019, and 54.5% of the patients (*N* = 4144) were diagnosed during the COVID-19 pandemic from 2020 to 2021.

About 32.1% of the patients (*N* = 2440) had DKA at the time of manifestation. There was a slight but significant difference (*p*  < 0.001) for HbA1c at diagnosis comparing the pre and postpandemic groups. Before the pandemic, mean HbA1c, adjusted for sex, age, BMI-SDS, migration, DKA, and AID use, was 11.21%, during the pandemic it was 11.40% with a mean estimated difference of 0.19% [0.1; 0.3].

**Figure 2:**
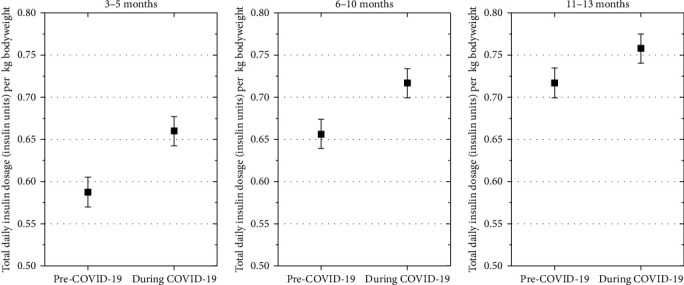
The estimated mean weight-adjusted insulin requirement was higher in patients with diabetes onset during the COVID-19 pandemic compared to those with diabetes onset before the pandemic. Total daily insulin dosage (insulin units) per kg bodyweight at three follow-up time-points. Estimates are adjusted for age at onset, gender, immigrant background, DKA at onset, and AID.

The estimated mean weight-adjusted insulin requirement was higher in patients with diabetes onset during the COVID-19 pandemic compared to those with diabetes onset before the pandemic at 3–5 months (mean estimated difference, 0.06 U/kg [95% CI 0.04; 0.07], *p*  < 0.001), at 6–10 months (mean estimated difference, 0.05 U/kg [0.03; 0.06], *p*  < 0.001), and at 11–13 months (mean estimated difference, 0.03U/kg [0.01; 0.05], *p*  < 0.001) after type 1 diabetes diagnosis (Figure 2).

**Figure 3:**
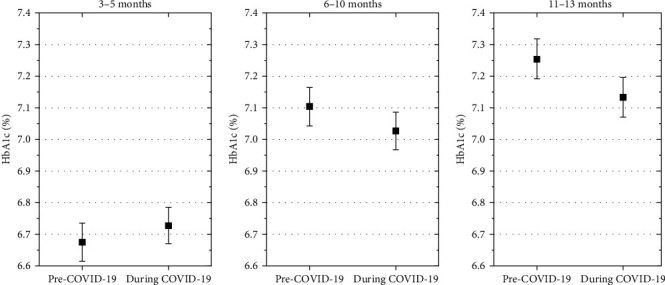
Estimated mean HbA1c 3–5 months after diagnosis was not significantly higher in children diagnosed with diabetes during the COVID-19 pandemic that in those diagnosed before, 6–10 months and 11–13 month after diagnosis, the estimated mean HbA1c in the pandemic onset group were lower than in the prepandemic onset group. HbA1c% in the two cohorts at three follow-up time-points, estimates are adjusted for age at onset, gender, immigrant background, DKA at onset, and AID.

Estimated mean HbA1c 3–5 months after diagnosis was not significantly higher in children diagnosed with diabetes during the COVID-19 pandemic than in those diagnosed before (mean estimated difference, 0.04% [−0.01; 0.08], *p*=0.11). In contrast, 6–10 months and 11–13 month after diagnosis, the estimated mean HbA1c in the pandemic onset group were lower than in the pre pandemic onset group (mean estimated differences, −0.1% [−0.1; −0.04], *p*  < 0.001; and −0.14% [ −0.2; −0.09], *p*  < 0.001, Figure 3).

**Figure 4:**
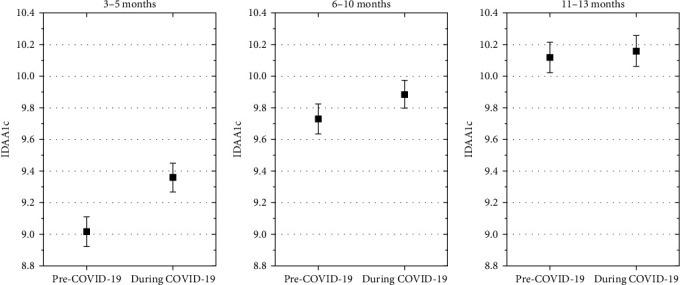
The estimated mean IDAA1C was significantly higher in children with onset of type 1 diabetes during the COVID-19 pandemic compared with those with diabetes onset before the COVID-19 pandemic at 3–5 months, but not at 6–10 months after diagnosis and at 11–13 months after diabetes diagnosis. IDAA1c in the two cohorts at three follow-up time-points. Estimates are adjusted for age at onset, gender, immigrant background, DKA at onset, and AID.

Adjusted for demographics, DKA at onset and AID, the estimated mean IDAA1C was significantly higher in children with onset of type 1 diabetes during the COVID-19 pandemic compared with diabetes onset before the COVID-19 pandemic at 3–5 months (mean estimated difference, 0.26% [0.17; 0.35], *p*  < 0.001), but not at 6–10 months after diagnosis (mean estimated difference, 0.08% [−0.01; 0.17], *p*=0.07), and at 11–13 months after diabetes diagnosis (mean estimated difference, −0.03% [−0.12; 0.7], *p*=0.6), (Figure 4).

**Figure 5:**
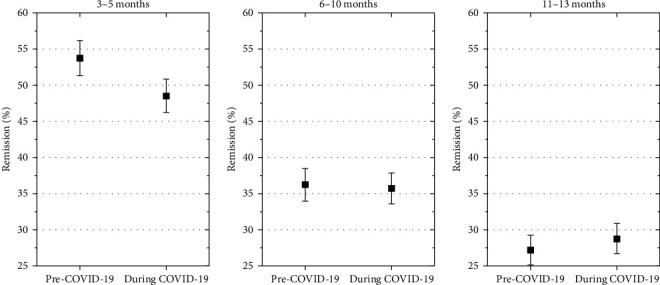
Remission was significantly less frequent in patients with diabetes diagnosis during the COVID-19 pandemic at 3–5 months after diagnosis, but not at 6–10 months and at 11–13 months. Rate of remission (IDAA1C ≤ 9%) based on adjusted IDAA1C in the two cohorts at three follow-up time-points. Estimates are adjusted for age at onset, gender, immigrant background, DKA at onset, and AID.

Remission was significantly less frequent in patients with diabetes diagnosed during the COVID-19 pandemic at 3–5 months after diagnosis (estimated odds ratio, 0.85 [0.77; 0.93], *p*=0.001), but not at 6–10 months (estimated odds ratio, 1.02 [0.92; 1.13], *p*=0.7), and at 11–13 months (estimated odds ratio, 1.13 [1.02; 1.30], *p*=0.03). (Figure 5).

## 4. Discussion

In this study, the remission in children and adolescents with new-onset type 1 diabetes occurred less frequently during the COVID-19 pandemic compared to the prepandemic period for the first 5 months after diabetes onset. It has been shown that IDAA1C, which is defined by the amount of insulin and the HbA1c that can be achieved by this insulin dose, correlates well with residual ß-cell function and can be used as a valid parameter to evaluate remission in children. In a previous publication from our group, we critically discussed the validity and limitations of IDAA1C as a surrogate marker for stimulated C-peptide to define remission of type-1 diabetes [13, 14, 18].

In our study, 3–5 months after diabetes diagnosis, only 48% of patients were in remission during the COVID-19 pandemic, compared to a remission rate of 53% before the pandemic in 2018/2019. In a publication from K. Nagl from 2017, using the multicenter DPV database from Austria and Germany, comparing data from 3657 children with new-onset T1D, who were continuously followed for up to 6 years, the prevalence of partial remission defined by IDAA1c ≤9 was 71% [18].

This could suggest a change in the autoimmune process during the COVID-19 pandemic, including a more rapid autoimmune destruction in patients with new-onset type 1 diabetes.

If we compare this report from 2017 with our data from 2018/2019, it has to be kept in mind that there was less pump use in the cohort described by Nagl et al. [18]. Pump use is associated with lower insulin requirement, therefore, the definition of remission is fulfilled more frequently in patients on insulin pumps. During recent years, there was a great progress in diabetes technology. Patients with insulin pumps need less insulin, which would speak in favor of more remission. HbA1c might be lower due to more diabetes technology use. Another important factor is increased BMI. We therefore, adjusted our data for DKA, pump and AID use, and BMI.

It is important to understand remission and particularly the factors influencing remission [19, 20]. Another risk factor for a lower rate of remission is the higher frequency of DKA in children with onset of diabetes during the pandemic [1, 2]. The delayed diagnosis of type 1 diabetes increases the risk for DKA and is associated with reduced residual insulin secretory capacity, and therefore, a shorter and less pronounced remission period. This hypothesis is supported by three findings: (1) HbA1c at onset was slightly, but significant (*p*  < 0.001) higher, (2) the frequency of severe DKA at onset was increased, and (3) the estimated mean weight-adjusted insulin requirement was higher in patients with diabetes onset during the COVID-19 pandemic compared with those with diabetes onset before the pandemic [1, 2].

Indirect effects that could have influenced the likelihood of remission are increased stress from the pandemic itself, from psychological burden, or from altered access to medical care. Social contacts, child health care practices, and the behavior of families abruptly changed during the COVID-19 pandemic, due to COVID-19 control efforts. Significant changes during the COVID-19 pandemic in lifestyle, eating habits, screen time, and physical activities have been shown in previous studies [21]. Therefore, the higher insulin requirement can partially be explained by lack of physical activities and more psychological burden. However, we could not detect an increase in BMI-SDS during the COVID-19 pandemic in our study. Besides socio-cultural aspects, the increased insulin requirement at follow-up could suggest a more rapid autoimmune progression during the pandemic [3]. Reduced microbial exposure due to increased hygiene practices during the pandemic may also have contributed to the observed decrease in remission of type 1 diabetes, as hypothesized by the hygiene hypothesis [10].

A more pronounced remission, both in length and in intensity, is associated with a more favorable long-term course of type 1 diabetes [19, 22–24]. Adult patients with type 1 diabetes and IDAA1C ≤ 9% showed less microvascular complications, as well as fewer episodes of severe hypoglycemia [23, 25]. Patients profit from a longer remission phase due to better glycaemic control and less risk of serious hypoglycemia in the first months. Currently, many studies address the remission phase, and medical interventions, such as the use of verapamil or teplizumab are tested, aiming to prolong the remission period [26]. Teplizumab, a humanized Fc-mutated anti-CD3 monoclonal antibody, was approved in the US in 2022 to delay progression to stage 3 type 1 diabetes [27, 28]. Children with shorter remission of type 1 diabetes experience a faster decline in beta cell function and face more difficulties in managing their disease, such as higher HbA1c levels and a higher risk of severe hypoglycemia [29].

A limitation of our study may result from the different circumstances and family resources that the children encountered at home during the COVID-19 pandemic, which may play a role in the treatment of young children with type 1 diabetes and could affect the amount of insulin needed. Several studies have highlighted the exacerbation of pre-existing inequities in diabetes management and outcomes. Valenzuela et al. [30] reported that household income was the most important predictor of glycemic health among racially/ethnically diverse youth with diabetes.

The strength of this investigation is the large amount of real-world data from a multinational multicenter registry, reflecting heterogeneity and current treatment in children with type 1 diabetes during the pandemic.

## 5. Conclusion

In summary, data from 7603 pediatric patients with new-onset of type 1 diabetes showed that the COVID-19 pandemic negatively affected the remission phase during the first 5 months after diabetes diagnosis.

The higher IDAA1C during the COVID-19 pandemic could suggest a more rapid autoimmune destruction in patients with new-onset of type 1 diabetes, delayed diabetes diagnosis, or increased psychosocial stress [3].

Lugar et al. [5] found that SARS-CoV-2 infection was temporally associated with the development of islet autoantibodies in young children with a high genetic risk for type 1 diabetes. Also, Rahmati et al. [31] conducted a systematic review and meta-analysis, which indicated that SARS-CoV-2 infection in children and adolescents was associated with a 42% higher risk of developing new-onset type 1 diabetes compared to non-COVID-19 control groups. This risk was particularly significant in children aged 0–11 years [31]. Boboc et al. [32] observed that children with positive SARS-CoV-2 serology had a higher percentage of detectable IA-2A antibodies and were more likely to be positive for multiple islet autoantibodies (GADA, ICA, and IA-2A) compared to those without SARS-CoV-2 serology. This suggests a potential link between SARS-CoV-2 infection and the onset of type 1 diabetes [32]. But to date, there is no reliable evidence that SARS-CoV-2 infections can cause autoantibody-negative diabetes by directly damaging ß-cells or autoantibody-positive type 1 diabetes by triggering autoimmunity [3].

Further studies are needed to analyze the underlying mechanisms and the long-term consequences of the COVID-19 pandemic on the autoimmune process in type 1 diabetes.

In order to elucidate the underlying mechanisms and the long-term consequences of the COVID-19 pandemic on the autoimmune process in type 1 diabetes, future population-based observational studies should include antibody/PCR-testing to objectively quantitate COVID-19 infection frequency together with standardized, validated questionnaires to reflect real-life burden in subjects with stages 1–3 of type 1 diabetes. PCR, IgM, and IgG titers will allow to discriminate between acute and previous viral infections, both of which might contribute to the pathophysiology of type 1 diabetes. We recommend PCR tests during the acute phase of infection (typically within the first few days after symptoms start or after exposure). For a postinfection monitoring, we propose antibody tests, which may be done approximately 1–3 weeks after the onset of symptoms or after a positive PCR test. A follow-up testing can be done at 1–3-month intervals [33].

The questionnaires that may be used measure various aspects, including health-related quality of life (HRQoL), diabetes-specific distress, and the impact of diabetes on daily living, for example: Diabetes Quality of Life Scale (DQOL), PedsQL Diabetes Module, Diabetes Distress Scale (DDS), and the WHO-5 Well-Being Index [34].

## Figures and Tables

**Table 1:** Children with new-onset type 1 diabetes in 2018–2019 (pre-COVID-19) vs 2020–2021 (during COVID-19).

Variable	Diabetes duration																			
	At manifestation					3–5 months					6–10 months					11–13 months				
	Pre-COVID-19		During COVID-19			Pre-COVID-19		During COVID-19			Pre-COVID-19		During COVID-19			Pre-COVID-19		During COVID-19		
	2018–2019		2020–2021			2018–2019		2020–2021			2018–2019		2020–2021			2018–2019		2020–2021		
	*N*	Mean (StD)	*N*	Mean (StD)	*p*-Value	*N*	Mean (StD)	*N*	Mean (StD)	*p*-Value	*N*	Mean (StD)	*N*	Mean (StD)	*p*-Value	*N*	Mean (StD)	*N*	Mean (StD)	*p*-Value
Weight-SDS	3319	0.04 (1.07)	4015	0.07 (1.07)	1.0	3369	0.29 (0.97)	4056	0.33 (0.99)	0.24	3411	0.31 (0.96)	4090	0.35 (0.98)	0.46	3375	0.35 (−0.96)	4026	0.38 (0.98)	0.74
Height-SDS	3319	0.31 (1.11)	4015	0.38 (1.10)	0.01	3369	0.21 (1.05)	4056	0.29 (1.05)	0.00	3411	0.21 (1.04)	4090	0.29 (1.04)	0.00	3375	0.22 (1.04)	4026	0.29 (1.03)	0.01
BMI- SDS	3319	−0.18 (1.19)	4015	−0.19 (1.19)	1.0	3369	0.26 (0.98)	4056	0.25 (1.01)	1.00	3411	0.29 (0.96)	4090	0.28 (1.01)	1.00	3375	0.34 (0.97)	4026	0.33 (1.00)	1.00
Pump (%)	3461	27.48	4142	30.83	0.02	3461	32.51	4142	35.73	0.02	3461	38.28	4142	43.46	<0.001	4142	40.39	4142	46.62	<0.001
Sensor (%)	3461	35.94	4142	51.88	<0.001	3461	70.1	4142	86.89	<0.001	3461	80.06	4142	91.98	<0.001	4142	80.12	4142	90.83	<0.001
SAP (%)	3461	8.49	4142	15.35	<0.001	3461	22.07	4142	31.05	<0.001	3461	29.09	4142	39.35	<0.001	4142	32.16	4142	42.18	<0.001
AID (%)	3461	20.25	4142	21.92	0.76	3461	22.74	4142	24.38	0.46	3461	25.08	4142	28.34	<0.001	4142	26.38	4142	29.48	0.01
DKA (%)	3461	27.77	4056	35.68	<0.001	—	—	—	—	—	—	—	—	—	—	—	—	—	—	—

## Data Availability

Due to patient privacy protection, original patient-level data are not allowed to be shared with outside researchers. However, remote data access and joint projects are possible.
